# Food-web complexity, consumer behavior, and diet specialism: impacts on ecosystem stability

**DOI:** 10.1007/s12080-024-00580-w

**Published:** 2024-04-25

**Authors:** Tommi Perälä, Mikael Kuisma, Silva Uusi-Heikkilä, Anna Kuparinen

**Affiliations:** 1 Department of Biological and Environmental Science, University of Jyväskylä, Jyväskylä, Finland; 2 Department of Physics, Technical University of Denmark, Lyngby, Denmark

**Keywords:** Biomass oscillations, Species persistence, Ecological networks, Niche model, Metabolic theory, Allometric trophic network

## Abstract

Ecological stability is a fundamental aspect of food web dynamics. In this study, we explore the factors influencing stability in complex ecological networks, characterizing it through biomass oscillations and species persistence. Using an Extended Niche model, we generate diverse food web structures and investigate the effects of intraspecific consumer interference, network size, connectance, and diet specialism on stability. Our findings reveal that intraspecific consumer interference plays a pivotal role in shaping stability. Higher interference results in stable dynamics, reducing oscillations and extinctions. Additionally, differences emerge between food webs comprised of invertebrate consumers and those of ectotherm vertebrates, with the latter showing higher oscillations. Network size and connectance also influence stability, where larger and more connected webs tend to exhibit reduced oscillations. Overall, our study sheds light on the complex interplay of factors affecting ecological stability in food webs. Understanding these dynamics is crucial for biodiversity conservation and ecosystem management.

**Supplementary Information:**

The online version contains supplementary material available at 10.1007/s12080-024-00580-w.

## Introduction

The stability of natural populations and ecosystems has intrigued ecologists for decades, sparking both theoretical and empirical investigations (e.g., reviewed by Dunne et al. 2002; Brose et al. 2006; Landi et al. 2018). The application of classic local stability theory to early ecological models, such as those used by May (1974), suggests that increasing complexity in ecological networks, in terms of more species and interactions, could lead to instability (see e.g., Dougoud et al. 2018; Landi et al. 2018). This forms the basis of May’s diversity-stability paradox, where the theoretical expectation conflicts with the observed stability in real-world ecosystems. While some researchers have explored stability within small modules of a few interacting species (Huxel and McCann 1998; Emmerson and Raffaelli 2004; van Altena et al. 2016), comprehensive demonstrations of how large, complex, and ecologically realistic networks persist dynamically have remained limited and not fully understood (but see Haydon 2000; Allesina and Pascual 2008; Gross et al. 2009).

In this context, the work of Brose and colleagues (2006) offers valuable insights. They combined structural models of complex food webs with nonlinear bioenergetic models of population dynamics, using parameters that are allometrically scaled to populations’ average body masses. Their findings brought to light a crucial factor in ecological stability: predator-prey body mass ratios. They discovered that increasing predator-prey body mass ratios can enhance population persistence up to a saturation level. Remarkably, this saturation point aligns with empirical predator-prey body mass ratios observed in natural ecosystems. Furthermore, they revealed that the negative effects of diversity, such as species richness, on stability (i.e., population persistence) can become neutral or even positive at these empirical predator-prey body mass ratios. These findings emphasize the significance of predator-prey body mass ratios in enabling the persistence of populations within complex food webs and stabilizing the diversity of natural ecosystems.

Rall et al. (2008) conducted extensive analyses of food webs, demonstrating the effects of complexity, omnivory, and interaction strength on stability. Their investigation centered on resolving the “paradox of enrichment” in consumer-resource systems with Holling type II, Holling type III, and Beddington-DeAngelis predator-interference functional responses. They demonstrated that consumer-resource systems with the last two are highly robust against accelerated oscillations due to enrichment. In a similar vein, Rip and McCann (2011) reviewed consumer-resource theory, highlighting the paradox of enrichment as a special case of a broader theoretical result. They argued that increased energy flux, relative to the consumer loss rate, contributes to top-heavy interactions and decreased stability.

However, one crucial aspect still eludes a comprehensive explanation: the drivers of population oscillations within complex food webs. Given the intrinsic interdependence of stability, particularly in terms of species persistence, and dynamics in ecological systems, it is essential to understand biomass fluctuations in stabilized food webs. Rall et al. (2008) provide valuable insights into this relationship, demonstrating a negative correlation between species persistence and the magnitude of oscillations in their study. In practice, fluctuations can arise from various sources, including external environmental drivers (Kuparinen et al. 2019) and intrinsic factors linked to food web dynamics (Brose et al. 2006). These intrinsic factors can also encompass over-compensatory density-dependent dynamics and cohort resonance in marine fish, both of which can be closely linked to food availability early in life (Bjørnstad et al. 2004; Botsford et al. 2014).

In this context, it is essential to recognize that predator and prey oscillations are known phenomena in ecological systems. For instance, in the Canadian boreal forest, lynx populations surge roughly every decade, followed by rapid declines in hare populations (Stenseth et al. 1997). Aquatic ecosystems provide additional examples, with predator-prey oscillations evident in zoo- and phytoplankton communities within lakes (Scheffer et al. 1997). Sockeye salmon populations, vital both economically and ecologically, exhibit remarkable population oscillations (Ricker 1997), with the nursery lake’s predator-prey interaction emerging as a plausible explanation for these oscillations (Guill et al. 2011; Schmitt et al. 2016).

Early theoretical work with simplified food web models suggested that omnivory by top predators or weak interactions could potentially dampen population oscillations (McCann and Hastings 1997; McCann et al. 1998). Additionally, Oksanen et al. (2001) proposed that diet specialism might be linked to oscillatory biomass dynamics due to differing functional responses between specialist and generalist predators. Empirical studies have also hinted at the role of specialization in driving oscillations (Erlinge et al. 1983; Hanski et al. 1991). For example, in regions with rodent specialists, such as northern Fennoscandia, owl populations heavily depend on rodent prey, resulting in pronounced oscillations unlike opportunistic rodent predators (Andersson and Erlinge 1977). A modeling study by Benincà and colleagues (2009) further illustrates how specialist predator species can perpetuate alternations in species dominance.

In this study, we aim to build upon the insights provided by Brose et al. (2006) and other researchers by delving into the correlates of biomass oscillation within complex food webs. One of our primary objectives is to study how diet specialism, characterized by the niche range width in the Niche model (Williams and Martinez 2000), affects biomass oscillations. To achieve this goal, we explicitly modify the traditional Niche model, resulting in a novel 3-parameter Extended Niche model, which allows us to create a wider range of food web topologies than previously possible. Specifically, our modification generalizes the distribution of niche range widths to account for a more variable distribution. Our analysis focuses on four key factors: diet specialism (Oksanen et al. 2001), metabolic type (Brose et al. 2006), intraspecific consumer interference (Rall et al. 2008), and complexity (reviewed by Landi et al. 2018), as reflected by species number and connectance. By exploring these elements with our modified Niche model combined with Allometric Trophic Network (ATN) dynamics, we seek to provide valuable insights that expand our understanding of the intricate dynamics at play within ecological systems.

## Material and methods

### Extended Niche model

In the standard 2-parameter Niche model, ${{\text{NICHE}}}_{2}(S, C)$, where $S$ is the number of species or species richness, and $C$ is the desired network connectivity (Williams and Martinez 2000), each species is first randomly assigned a niche value ${n}_{i}$ from the uniform distribution ${\text{Uniform}}(0, 1)$. The niche range width is then randomly drawn for each species as ${R}_{i}={n}_{i}{X}_{i}$, where ${X}_{i} \sim {\text{Beta}}(\alpha , \beta )$, and $\alpha =1$ and $\beta =(1-2C)/2C$. The center of the consumer’s niche range is then randomly drawn as ${m}_{i} \sim {\text{Uniform}}(\frac{{R}_{i}}{2}, {n}_{i})$. Finally, the network topology is determined by creating feeding links between species $i$ and $j$ if the niche value of the resource $j$ is in the niche range of the consumer $i$, i.e., ${n}_{j}\in \left[{m}_{i}-\frac{{R}_{i}}{2}, {m}_{i}+\frac{{R}_{i}}{2}\right]$.

One of our aims is to study how diet specialism, which is characterized by the niche range width ${R}_{i}$ in the Niche model, affects biomass oscillations in the food web dynamics. Increasing $C$ naturally increases the average number of resource species per consumer but increasing $C$ acts on the entire food web, and thus, it is not an effective way of controlling the amount of specialism in the food web. Our goal now is to generalize the distribution of ${X}_{i}$ beyond ${\text{Beta}}(1, \frac{1-2C}{2C})$ to account for more variable distribution of niche range widths.

To this end, we take the simplest possible extension, and begin also utilizing the $\alpha$ parameter in the beta distribution. First, we redefine new coordinates for beta-distribution parameters $(\alpha , \beta )$. We take one coordinate as $C$, and choose the other coordinate, denoted as $\chi$ such that

1 $$\begin{array}{c}\mathrm\alpha\;=\;\mathrm\chi\;\text{cos}\;\mathrm{atan}\frac{1\;-\;2\mathrm C}{2\mathrm C}\\\mathrm\beta\;=\;\mathrm\chi\;\text{sin}\;\mathrm{atan}\frac{1\;-\;2\mathrm C}{2\mathrm C}.\end{array}$$

**Fig. 1:**
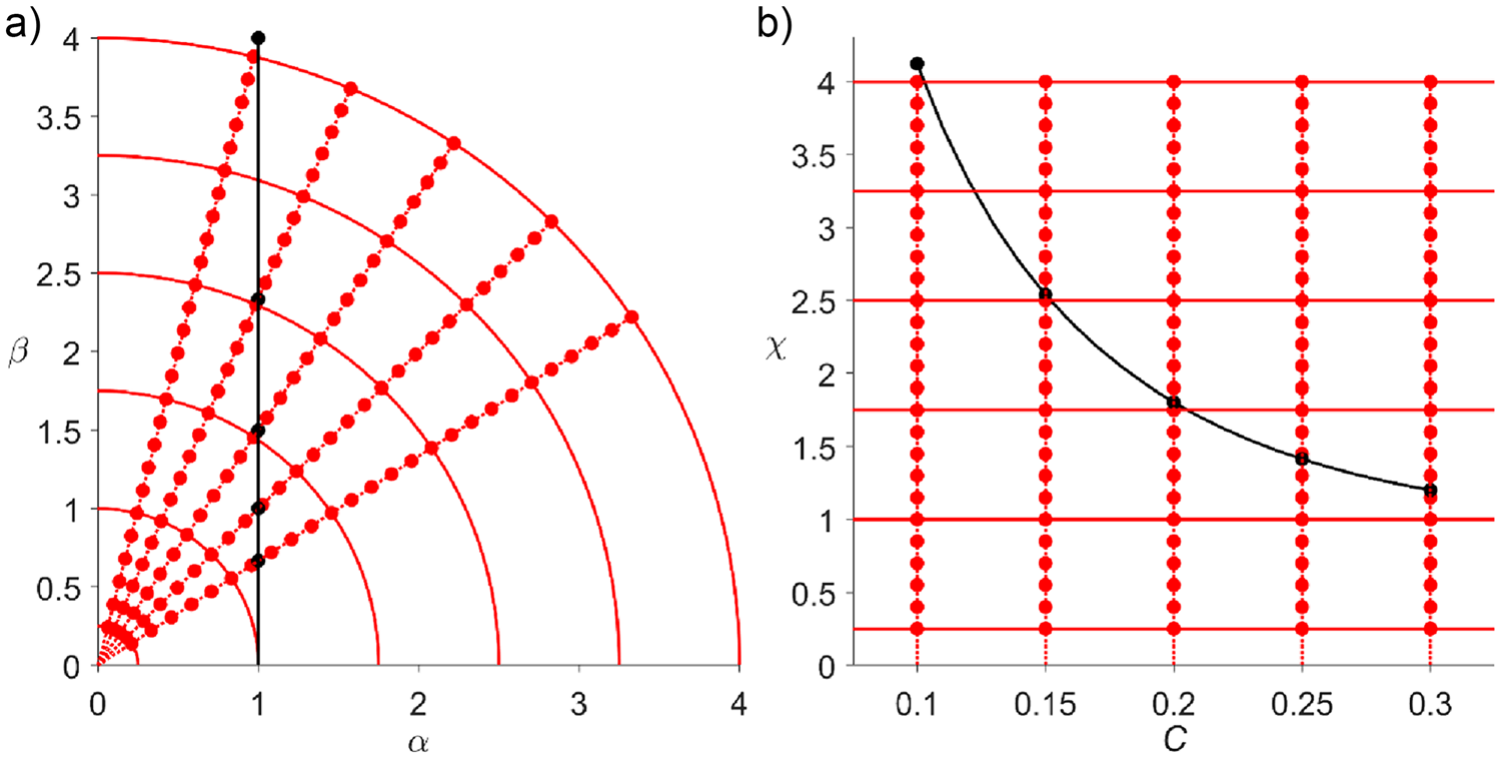
Illustration of the curvilinear coordinate system for the parameters of the 3-parameter Extended Niche model, ${{\text{NICHE}}}_{3}(S, C, \chi )$. **a** Red solid curves denoting constant $\chi$ values, and red dotted lines denote constant $C$ values. The black line represents the possible $(\alpha , \beta )$ values obtained by the standard 2-parameter Niche model, ${{\text{NICHE}}}_{2}(S, C)$, with black dots indicating $C\in \left\{0.10, 0.15, 0.20, 0.25, 0.30\right\}$. The red dots depict the grid of $C$ and $\chi$ values used in our simulations. **b** The parameters are displayed in the $(C, \chi )$ coordinates. Notably, the original 2-parameter Niche model, assuming $\alpha =1$ and $C$ within an ecologically justifiable interval [0.1, 0.3], corresponds to $\chi$ values roughly ranging from 1.25 to 4.2. This alignment provides ecological justification for the explored range of $\chi$ values in our study

This creates a curvilinear coordinate system, where $C$ and $\chi$ are orthogonal in $(\alpha , \beta )$. In Fig. 1, this coordinate transformation is illustrated graphically. This results in our 3-parameter Extended Niche model, ${{\text{NICHE}}}_{3}(S, C, \chi )$. Except for the $\alpha$ and $\beta$ values for the beta-distribution, the algorithm for generating the food-web structure is identical to the original Niche model.

To gain deeper insights into the impact of the new parameter $\chi$ on the Extended Niche model, we conducted an analysis focusing on the distribution of niche range widths under various scenarios (Fig. S1). Notably, the influence of $\chi$ on niche range widths is evident; higher values of $\chi$ result in distributions that exhibit reduced right-skewness. This observation is supported by visual cues such as the rightward shift of the median (mean remaining constant by definition) and a simultaneous decrease in the nonparametric skewness, calculated as the difference between the mean and the median, divided by the standard deviation.

### Generating food web topologies

We used the Extended Niche model ${{\text{NICHE}}}_{3}(S, C, \chi )$ to generate food webs of size $S\in \left\{10, 20, 30\right\}$ and expected connectance $C\in \left\{0.10, 0.15, 0.20, 0.25, 0.30\right\}$. We also varied $\chi$ by choosing 26 equally spaced values (with increments of 0.15) from the interval [$0.25, 4.00]$. This range aligns with ecologically justifiable values based on the considerations of the original 2-parameter Niche model, assuming $\alpha =1$ and $C$ within an interval [0.1, 0.3], corresponding to $\chi$ values roughly ranging from 1.25 to 4.2 (Fig. 1). Overall, we set out to generate food webs in a full factorial manner with $3\times 5\times 26=390$ different parameter combinations for $\left(S, C, \chi \right)$.

For each parameter combination, we aimed to generate 1000 random food web topologies. We required that the realized connectance of the food web was within $\pm 3\%$ of the expected connectance. We also required that 20–30% of the species in the food web were designated as primary producers, i.e., did not have any resource species on their niche range. Moreover, the graph representing the food-web topology had to be acyclic (without cycles or loops) and weakly connected. The acyclicity requirement ensured that cannibalism was not present in the food webs. This decision was guided by our study’s focus on intraspecific feeding interference; we aimed to avoid introducing another intraspecific interaction in the model that could confound its effects. Weak connectedness was essential to avoid non-connected food webs, which would result in compartments that do not interact in any way. Furthermore, we checked that no two graphs were isomorphic to ensure that each food web had a unique structure. This was accomplished by first checking if the most recently generated food web had a unique sorted prey-averaged trophic position vector (Eq. 2), in which case the food web was also unique. If this first test failed, we next compared the standard deviations of the outdegree distributions of the webs that had identical sorted trophic positions. If a generated web failed both negative tests, we ran a brute force algorithm that tested all possible perturbations of the food web that preserved the sorted trophic position vector to see if there existed an isomorphic mapping from the new food web to the existing food webs that had the same sorted trophic positions and the same standard deviation of the outdegree distribution. For some parameter combinations, particularly, $C=0.10$ and $\chi \le 0.55$, generating food webs that satisfy the specific condition—where basal producers constitute 20–30% of the species in the food web—proved to be extremely difficult. This challenge arises because small $\chi$ values lead to niche range width distributions where a greater proportion of the probability mass is concentrated closer to zero (Fig. S1). This, in turn, leads to a significant increase in the number of basal producers, and only rare outlier cases satisfy the set condition. Thus, we limited the number of attempts for any replicate of a given parameter combination to 10,000. If no valid food webs were found within 10,000 attempts, we proceeded to the next replicate. The number of food webs found for the problematic corner of the parameter space is shown in Table 1.

**Table 1:** The number of food webs found using the Extended Niche model for the problematic corner of the parameter space ($C=0.10$ and $\chi \le 0.55$)

$S$ \ $\chi$	0.25	0.40	0.55
10	354	963	1000
20	9	344	984
30	2	67	737

To summarize the impact of the $\chi$ parameter on key structural properties of the generated food webs, we examine its influence on the in-degree distribution (Fig. S2), the out-degree distribution (Fig. S3), and the average prevalence of specialists—defined as species with at least one but at most $0.1S$ resource species (Fig. S4). Notably, the effect on the in-degree distribution, serving as a proxy for vulnerability within the food webs generated by the Extended Niche model, was observed to be less pronounced. This indicates that other factors, such as network connectance, might play a more dominant role. The out-degree distribution, depicting alterations in generality across different scenarios of network connectance and $\chi$ values, demonstrated a subtle increase in the median with higher $\chi$ values—particularly evident in food webs with higher connectance. Moreover, across varying numbers of species and levels of network connectance, an increase in $\chi$ generally leads to a decrease in the number of specialists.

### ATN model for the food web dynamics

After establishing the food web link structure, the prey-averaged trophic positions $({{\text{TP}}}_{i}, i=1\dots S)$ of the nodes were solved from a following set of linear equations:

2 $${{\text{TP}}}_{i}\;=\;1\;+\;{\sum }_{j}{\omega }_{ij}{{\text{TP}}}_{j}$$

where we use equal resource preference parameters ${\omega }_{ij}=1/{N}_{i}$ if $i$ eats $j$, and zero otherwise, and ${N}_{i}$ is the number of resource species of species $i$. In other words, trophic position 1 was assigned to the basal producers (species having no resources), and trophic positions for each consumer species were calculated as the weighted average of the trophic positions of its resources plus one. The individual body size (body mass in micrograms of carbon) of each species was calculated by utilizing its trophic position as

3 $${M}_{i}\;=\;{Z}^{{{\text{TP}}}_{i}},$$

where $Z$ is the consumer-resource body size ratio. We explored the dynamics of food webs consisting of two different metabolic types (${\text{MType}}$): ectotherm vertebrates $(Z=100)$ and invertebrate consumers $(Z=10)$. The information about the body masses was then used to calculate the mass-specific metabolic rates of the consumer species using the allometric scaling relationship:

4 $${x}_{i}\;=\;{{a}_{x}M}_{i}^{-{b}_{x}},$$

where the allometric scaling exponent ${b}_{x}=-0.25$ and the allometric scaling coefficients ${a}_{x}=0.88$ and ${a}_{x}=0.314$ for ectotherm vertebrates and invertebrate consumers, respectively (Brose et al. 2006).

Functional response is an idealization of consumer’s consumption rates with respect to its resource densities, and possibly competitive interactions. In this study, we utilize the following DeAngelis-Beddington-Holling type III hybrid functional response:

5 $${F}_{ij}\left({\varvec{B}}\right)\;=\;\frac{{\omega }_{ij}{B}_{j}^{q}}{{B}_{0}^{q}\;+\;d{B}_{0}^{q}{B}_{i}\;+\;\sum_{k}{\omega }_{ik}{B}_{k}^{q} },$$

where ${\varvec{B}}$ denotes the vector of all species biomasses. The biomass of the focal resource is ${B}_{j}$ and $q=1.2$ is a form factor of the functional response. The “half-saturation density” ${B}_{0}=0.5$ and the biomass of the consumer is ${B}_{i}$. To study the effects of the consumer’s intraspecific feeding interference, $d$, on the food web stability, we tested 30 logarithmically distributed values on the interval $[0.01, 10]$. The functional response is utilized in the biomass dynamics of a consumer species $i$ as follows:

6 $${\dot{B}}_{i}\;=\;-\;{x}_{i}{B}_{i}\;+\;\sum_{j}y{x}_{i}{F}_{ij}\left({\varvec{B}}\right){B}_{i}\;-\;\sum_{k}\frac{y{x}_{k}{F}_{ki}\left({\varvec{B}}\right){B}_{k}}{{e}_{i}},$$

where ${\dot{B}}_{i}$ is the time derivative of ${B}_{i}$, and $y=4$ and $y=8$ (Brose et al. 2006) are the maximum feeding rate scaling factor for ectotherm vertebrates and invertebrate consumers, respectively. The assimilation efficiency, ${e}_{i}$, was determined based on the type of the resource (Brose et al. 2006):

7 $${e}_{i}\;=\;\left\{\begin{array}{cc}0.85,& \mathrm{if \;}i\;\mathrm{ is\ a\ consumer}\\ 0.45,& \mathrm{if \;}i\;\mathrm{ is\ a\ producer}.\end{array}\right.$$

The biomass dynamics of a producer species is modelled as

8 $${\dot{B}}_{i}\;=\;{r}_{i}\left(1\;-\;\frac{{B}_{i}\;+\;\sum_{j\in {I}_{{\text{p}}}}{B}_{j}}{K}\right){B}_{i}\;-\;\sum_{k}\frac{y{x}_{k}{F}_{ki}\left({\varvec{B}}\right){B}_{k}}{{e}_{i}},$$

where the mass-specific intrinsic growth rate of the producers was set to ${r}_{i}=1$, and the set of producer species indices is denoted by ${I}_{{\text{p}}}$. We set $K=100$ to determine the carrying capacity for the producers. It is important to clarify that $K$ acts as a parameter influencing the carrying capacity rather than representing the carrying capacity itself. Including ${B}_{i}$ twice in the numerator induces greater intraspecific competition.

### Conducting the simulations

For each simulation, we drew the initial biomasses ${B}_{i}(0)$ from a uniform distribution ${\text{U}}\left(0, 0.01\right)$. The system was then first simulated for a burn-in period of 100,000 time units to reach equilibrium, after which the dynamics for the next 10,000 time units were recorded. The integration was carried out using a Runge-Kutta method with a variable time step for efficient computation, implemented in MATLAB’s ode45 function.

The simulations were conducted in a swarm computing environment called the Decentralized Distributed Computing Environment for Matlab (DiCE.m, unpublished) utilizing approximately 480 CPUs on 12 different computers. All in all, approximately 23.11 million food web simulations were conducted. All codes and figures were produced in Matlab (MATLAB 2021).

### Quantifying the food web oscillations

Before examining the oscillations in the simulated biomasses, we implemented a procedure to identify and remove extinct species. Initially, we excluded those species whose biomass fell below the smallest positive normalized floating-point number. Subsequently, recognizing that the initial biomasses of species are arbitrary, and our primary interest lies in the equilibrium state or stable oscillation, we allowed the system sufficient time to reach these desired states. A simple criterion based on a low biomass threshold may not be effective, as species could potentially recover after an initial decline and reach a biomass within acceptable limits for persistence.

To address this, we developed an approach to determine if a species truly goes extinct in the simulation. Specifically, we iteratively removed species with the smallest biomass until the slope of the average logarithmic biomass of the species over time met a predefined threshold of -10^-4^. By examining the slope of the average biomass, we identified species exhibiting an exponential decay towards zero. We continued this process until this exponential decay was no longer observed, allowing us to identify the set of species that did not go extinct. Importantly, we intentionally avoided setting a lower limit for the biomass of a species; if a species could persist, it was considered in our analyses. The typical biomasses of the least abundant species remaining after the described procedure are summarized in Table S1.

After filtering out the extinct species using the above criteria, we calculated two measures of oscillation for the food web from the biomasses of the remaining ${S}^{*}$ species. First, we calculated the average of the differences between the maximum and the minimum of the common logarithm of species biomasses,

9 $${O}_{{\text{indiv}}}\;=\;\frac{1}{{S}^{*}}\sum_{i}\left(\underset{t}{{\text{max}}}\left({{\text{log}}}_{10}\;{B}_{i}\left(t\right)\right)\;-\;\underset{t}{{\text{min}}}\left({{\text{log}}}_{10}\;{B}_{i}\left(t\right)\right)\right)$$

Given that we have removed extinct species and conducted a sufficiently long burn-in to drive the system to equilibrium or oscillations around the equilibrium, this metric measures the extent of oscillation as defined by the difference between the crest and base of the wave on a logarithmic scale. This gives us information about how many orders of magnitude on average the individual species biomasses oscillate. Secondly, we calculated the coefficient of variation of the sum of the species biomasses to study the oscillation in the total biomass of the food web,

10 $${O}_{{\text{tot}}}\;=\;{\text{CV}}\left(\sum\limits_{i\;=\;1}^{{S}^{*}}{B}_{i}\left(t\right)\right)\;=\;\frac{{\text{std}}\left(\sum_{i\;=\;1}^{{S}^{*}}{B}_{i}\left(t\right)\right)}{{\text{mean}}\left(\sum_{i\;=\;1}^{{S}^{*}}{B}_{i}\left(t\right)\right)}.$$

While Eq. 9 focuses on the average individual species oscillation in orders of magnitude, Eq. 10 examines the coefficient of variation in the total biomass dynamics. Using both measures for oscillation not only provides a clearer understanding but also allows for a more refined analysis. For instance, if two species were to oscillate in opposite phases, examining the total biomass might suggest no overall oscillation. However, by separately analyzing each species and then averaging their measures of oscillation, we could reveal subtle oscillatory patterns that might be obscured at the aggregate level.

## Results

**Fig. 2:**
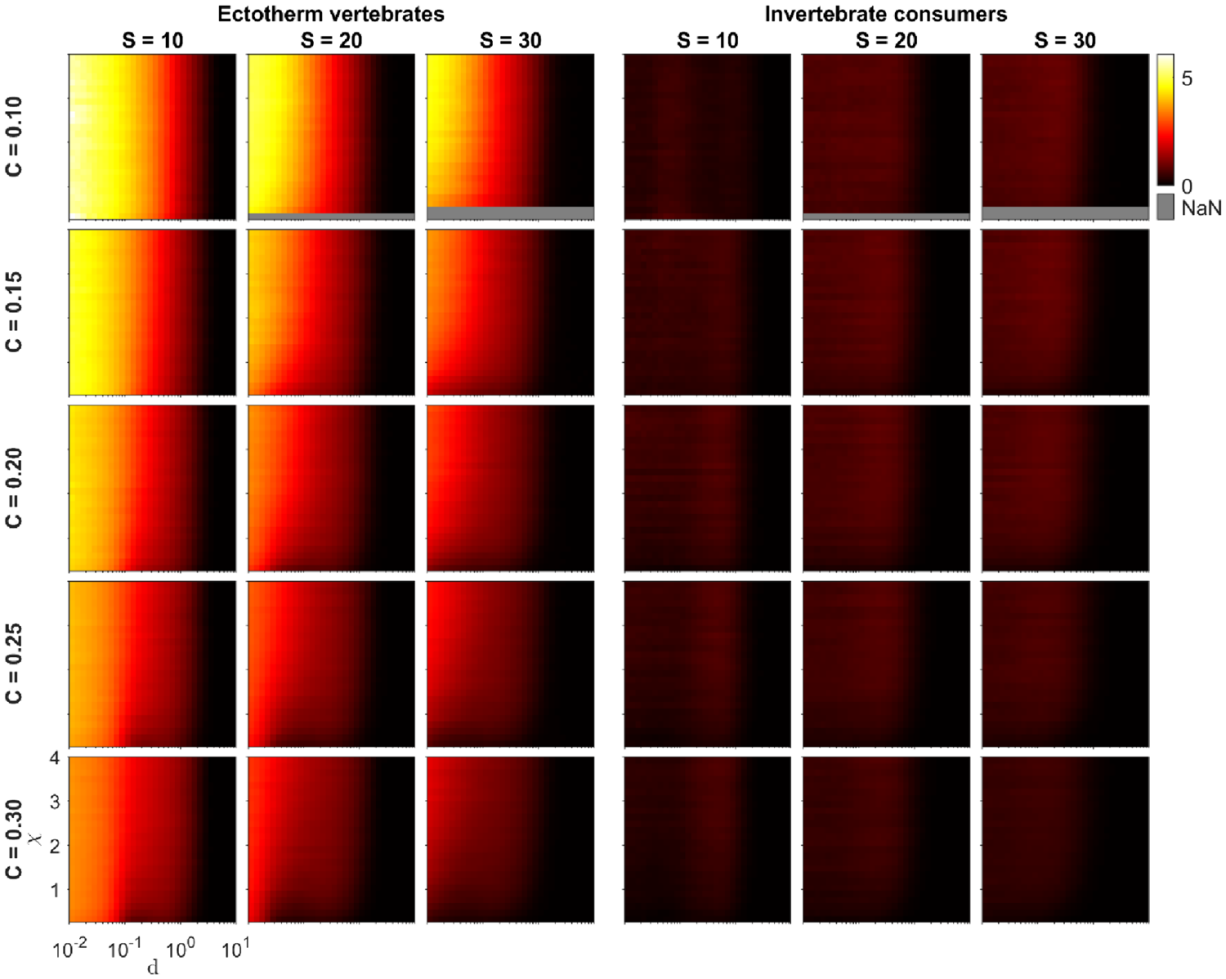
Food-web oscillation measure ${O}_{{\text{indiv}}}$ as a function of the second Extended Niche model parameter $\chi$ and the intraspecific consumer interference parameter $d$ for small, intermediate, and large network sizes $S$, five values of network connectance $C\in \{0.10, 0.15, 0.20, 0.25, 0.30\}$, and two metabolic types of consumers (ectotherm vertebrates, invertebrate consumers). The lighter the color, the higher the average ${O}_{{\text{indiv}}}$ for the food webs generated with the corresponding parameter value 5-tuple $({\text{MType}}, S, C, \chi , d)$. For the figure, we filtered out those parameter combinations that had less than 300 replicate food webs

**Fig. 3:**
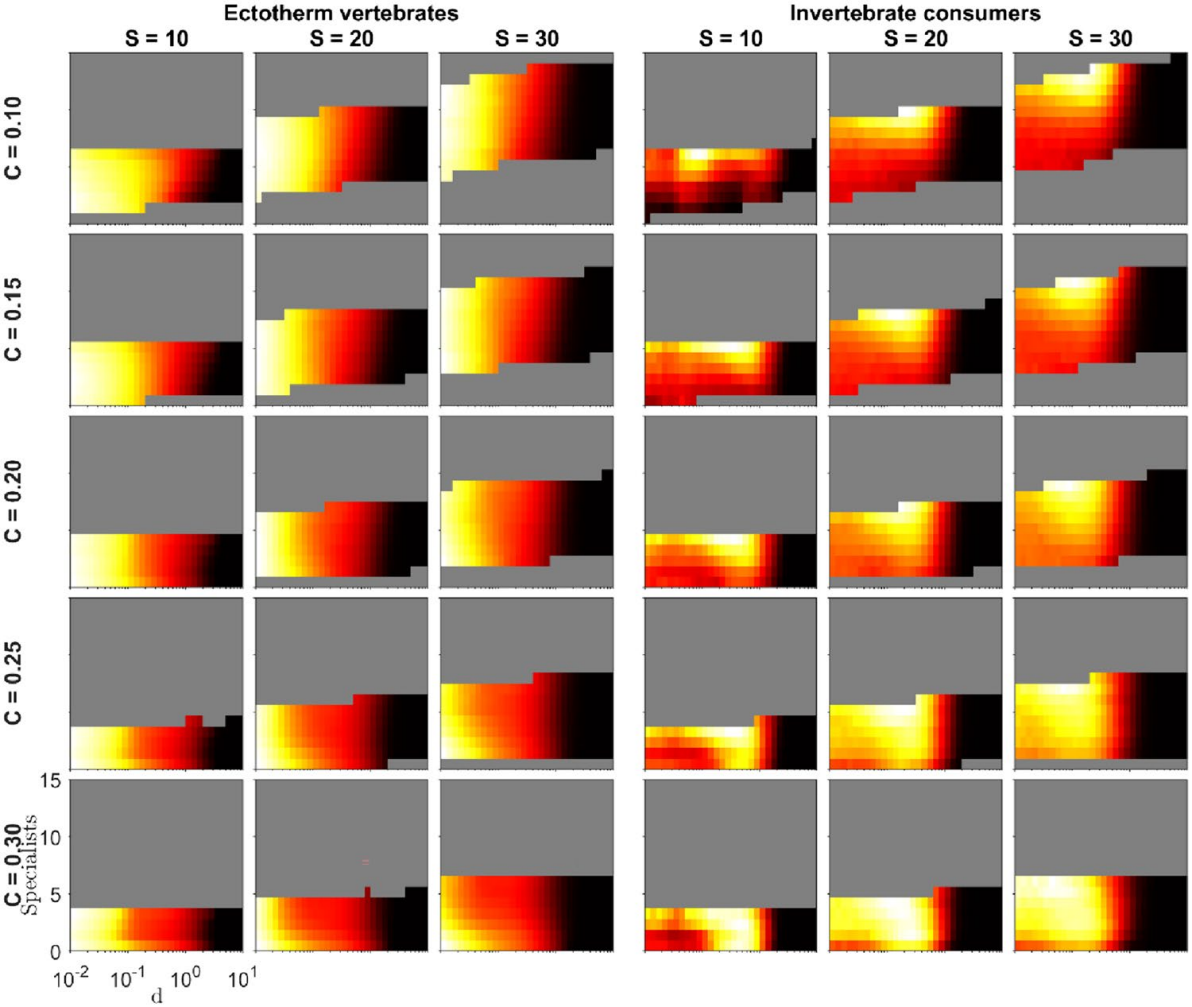
Food-web oscillation measure ${O}_{{\text{indiv}}}$ as a function of the number of specialists and the intraspecific consumer interference parameter $d$ for small, intermediate, and large network sizes, five values of network connectance $C\in \{0.10, 0.15, 0.20, 0.25, 0.30\}$, and two metabolic types of consumers (ectotherm vertebrates, invertebrate consumers). The lighter the color, the higher average ${O}_{{\text{indiv}}}$ for the corresponding food-web property-food-web dynamics parameter value pair $({\text{Specialists}}, d)$ within the panel. A species was considered a specialist when it had at most 10% of the species in the original food web on its diet. Each panel has its own scale, and as the figure attempts to highlight the relative effect of specialism within each panel, the color bars are not shown. Moreover, only those $({\text{Specialists}}, d)$ pairs are shown that had data from at least 700 simulations. Furthermore, for aesthetic reasons, a robust mean estimator was used, which ignored outliers whose distance to the mean was more than 4 standard deviations

The most significant factor influencing food web oscillations was the intraspecific consumer interference parameter $(d)$. Remarkably, high values of $d$ resulted in entirely stable dynamics with no oscillations, irrespective of other food web properties (Figs. 2 and 3, S1 and S2). The second most significant factor affecting oscillations when measured using ${O}_{{\text{indiv}}}$ was the metabolic type $({\text{MType}})$. Oscillations were considerably more pronounced in food webs comprising ectotherm vertebrates compared to those with invertebrate consumers (Fig. 2). In contrast, invertebrate consumer food webs exhibited minimal oscillations in comparison.

Food web size $(S)$ also played a substantial role, with oscillations consistently diminishing as $S$ increased. A similar effect was observed concerning connectance $(C)$, as oscillations were most pronounced in smaller $C$ values and notably decreased as $C$ increased. Interestingly, the $\chi$ parameter of the Extended Niche model also influenced oscillation magnitude in ectotherm vertebrate food webs. In general, reducing $\chi$ resulted in fewer oscillations.

The number of specialists in the food web had varying effects on oscillations, depending on other food web properties (see Fig. 3). For invertebrate consumer food webs, increasing diet specialism consistently led to higher oscillations. In ectotherm vertebrate food webs, elevated diet specialism increased oscillations for less connected food webs $(C\le 0.15)$ with moderate to high values of the consumer interference parameter $d$. For food webs with intermediate connectance $(C=0.20)$, the impact of the number of specialists appeared less clear, except for larger $d$ values, which resembled the less connected food webs. Interestingly, for highly connected food webs $(C\ge 0.25)$, increased specialism even reduced oscillations for medium and large food web sizes.

When considering the oscillation of the entire food web $({O}_{{\text{tot}}})$, the effect of ${\text{MType}}$ closely mirrored its effect on ${O}_{{\text{indiv}}}$: invertebrate consumer food webs exhibited much fewer oscillations than ectotherm vertebrate food webs (Fig. S5). In invertebrate consumer food webs, $S$ and $d$ affected ${O}_{{\text{tot}}}$ similarly to ${O}_{{\text{indiv}}}$, although the relative decrease in ${O}_{{\text{tot}}}$ due to increasing $S$ was more pronounced. Conversely, increasing $C$ did not decrease ${O}_{{\text{tot}}}$ but instead caused a slight increase. The influence of the Extended Niche model parameter χ was more noticeable, generally resulting in an increase in ${O}_{{\text{tot}}}$ with higher χ values.

The effect of diet specialism on ${O}_{{\text{tot}}}$ closely resembled its impact on ${O}_{{\text{indiv}}}$ (Fig. S6). In invertebrate consumer food webs, the highest oscillation consistently occurred with the maximum number of specialists. In ectotherm vertebrate food webs, the effect was more variable and dependent on $C$ and $S$.

**Fig. 4:**
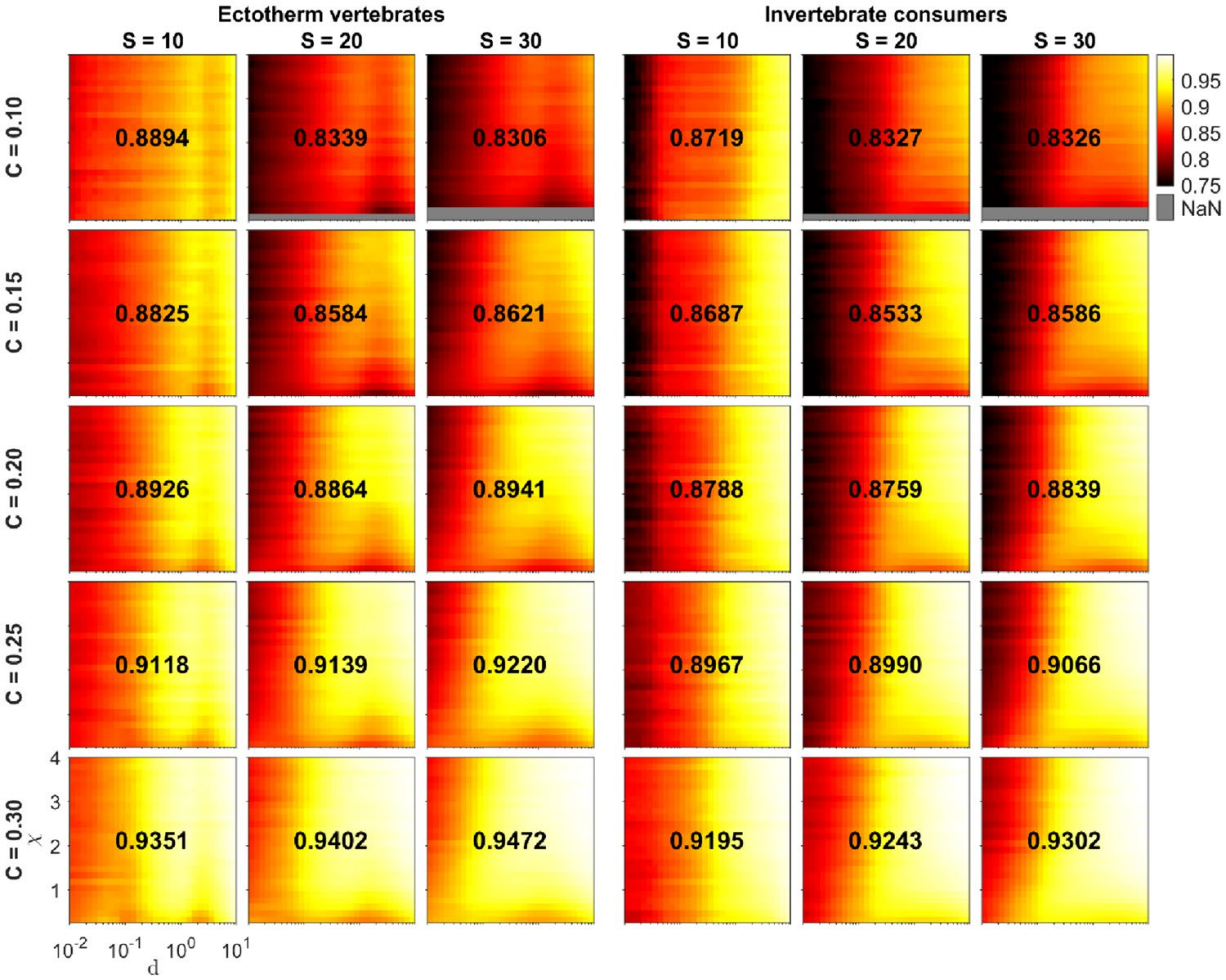
The fraction of persisting species as a function of the second Extended Niche model parameter $\chi$ and intraspecific consumer interference parameter $d$ for small, intermediate, and large network sizes $S$, five values of network connectance $C\in \{0.10, 0.15, 0.20, 0.25, 0.30\}$, and two metabolic types of consumers (ectotherm vertebrates, invertebrate consumers). The lighter the color, the higher the average fraction of persisting species in the food webs with the parameter 5-tuple $({\text{MType}}, S, C, \chi , d)$ after the burn-in period of 100,000 time units. The average of the average fractions of each 3-tuple $({\text{MType}}, S, C)$ is shown in the center of the panels. For the figure, we filtered out those parameter combinations that had less than 300 replicate food webs

We also examined the number of species persisting after extinctions during the 100,000-time unit burn-in period. The average fraction of persisting species ranged from 0.7210 to 0.9994 across various 5-tuple combinations $({\text{MType}}, S, C, \chi , d)$ (Fig. 4). Notably, the intraspecific consumer interference parameter $d$ emerged as the most influential predictor of species persistence, with higher $d$ values associated with greater species richness. For ectotherm vertebrate food webs, the overall average fraction of persisting species was slightly higher (0.8934) than for invertebrate consumers (0.8822).

For highly connected food webs $(C\ge 0.25)$, increasing food web size resulted in a higher average number of persisting species, whereas for the least connected food webs $(C=0.10)$, average species richness had a negative correlation with food web size. For intermediate connectance food webs $(0.15\le C\le 0.20)$, there was no clear trend. However, for large $(S=30)$ and intermediate $(S=20)$ food webs, average species persistence increased almost linearly with $C$, and small size food webs exhibited variations in this trend due to relatively high average species persistence for $C=10$ (Fig. S7). In terms of species persistence as a function of $S$ and $C$, both metabolic types exhibited similar behavior.

## Discussion

The findings of this study shed light on the complex interplay of factors that influence biomass oscillations in food webs. Within the range of the tested parameters, our results demonstrate that intraspecific consumer interference (parameter $d$) is the primary driver of stability within these ecological network models. Remarkably, high values of $d$ led to completely stable dynamics without any oscillations, regardless of other food web properties. This observation suggests that consumer interference, which accounts for competition among individuals of the same species, plays a pivotal role in shaping the stability of food webs.

Metabolic type $({\text{MType}})$ emerged as the second most significant factor affecting oscillations. Food webs composed of ectotherm vertebrates exhibited far greater oscillations compared to those comprising invertebrate consumers. This distinction highlights the importance of considering the metabolic characteristics of species when assessing food web stability. Ectotherm vertebrates, which include taxa like fish, may exhibit greater sensitivity to environmental fluctuations, potentially leading to more pronounced oscillations.

Our study also underscores the influence of food web size $(S)$ and connectance $(C)$ on oscillations. Larger food webs tended to exhibit smaller oscillations, suggesting that increased species richness and complexity may contribute to stability. Furthermore, higher connectance $(C)$ values were associated with reduced oscillations. These results emphasize the intricate relationship between food web structure and stability, with greater complexity and interconnectivity promoting overall resilience.

In our study, we uncovered a noteworthy impact of the $\chi$ parameter in the Extended Niche model on oscillations in ectotherm vertebrate food webs. Lower values of $\chi$, which result in niche range distributions concentrated closer to zero, were generally associated with reduced oscillations. This insight suggests that the distribution of niche range widths, influenced by $\chi$, can significantly impact food web dynamics.

The role of diet specialism in shaping oscillations proved to be complex and context dependent. In invertebrate consumer food webs, an increase in diet specialism consistently led to higher oscillations. However, in ectotherm vertebrate food webs, the effect varied depending on connectance $(C)$ and food web size $(S)$. This suggests that the influence of diet specialism on stability may be modulated by other factors, such as the presence of highly connected species.

We also examined species persistence during the simulation. Higher intraspecific consumer interference $(d)$ was associated with greater species richness. Ectotherm vertebrate food webs generally had slightly more persisting species compared to invertebrate consumers. The relationship between food web size $(S)$ and species persistence depended on connectance $(C)$, with highly connected food webs $(C\ge 0.25)$ and larger $S$ having more persisting species, while less connected food webs $(C=0.10)$ showed a decrease in species richness with increasing food web size. For food webs of intermediate connectance $(0.15\le C\le 0.20)$, there was no clear trend. Both metabolic types exhibited similar behavior regarding species persistence concerning $S$ and $C$.

Our study provides a distinctive perspective on food web dynamics compared to earlier works, specifically addressing the findings of Rall et al. (2008) and Rip and McCann (2011). Rall et al. (2008) in their study extensively explored the impact of enrichment by systematically varying the carrying capacity in consumer-resource interactions. They focused on the role of connectance and predator interference, which aligns with our consideration of intraspecific consumer interference. However, a notable distinction is that while Rall et al. (2008) investigated Holling type II, type III, and DeAngelis-Beddington functional responses separately, our study utilized a DeAngelis-Beddington-Holling type III hybrid functional response, offering a novel perspective to the study of food web stability. Similarly, Rip and McCann (2011) emphasized the destabilizing effects of increased energy flux in their study. They highlighted the top-heaviness of consumer-resource interactions, a perspective that contrasts with our focus on the complexity of food webs. Our study places specific emphasis on the magnitude of oscillations and species persistence, uncovering the intricate relationship between food web structure, consumer interference, and stability. This departure from traditional focuses on enrichment broadens our understanding of food web dynamics, offering insights into novel dimensions that collectively shape biomass oscillations and stability, along with species persistence.

While our study primarily focuses on intraspecific consumer interference as a key driver of stability, it is worth noting that the concept of near-neutral competition has gained prominence in recent ecological research. Rodríguez-Sánchez et al. (2020) explored the role of near-neutral competition in food web dynamics and found that it can increase the likelihood of developing chaotic dynamics, which in turn correlates with higher biodiversity. Our study extends these insights by demonstrating that intraspecific interference can also lead to increased stability by reducing oscillations in food web dynamics. Together, these findings highlight the intricate relationship between competition, oscillations, and biodiversity in ecological systems, offering new perspectives on the mechanisms driving complex food web dynamics.

A significant contribution of our study is the introduction of the Extended Niche model, which provides a unique framework to explore the dynamics of food webs. This extension of the celebrated Niche model by Williams and Martinez (2000) provides more flexibility and is able to generate a wider array of food web topologies, explicitly modifying the niche range width distribution by utilizing the new $\chi$ parameter (as demonstrated in Fig. S1) providing potential benefits for future food web studies.

The introduction of the Extended Niche model in our study also points to an intriguing avenue for future research in understanding how the new parameter, $\chi$, could be inferred from empirical food webs. While our study utilized a range of $\chi$ values between 0.25 and 4—encompassing empirically plausible values based on the considerations of the original niche model (see Fig. 1)—to examine its impact on food web dynamics, extracting $\chi$ from observed food web structures remains an open challenge. Probabilistic models like ours offer the possibility of calculating the likelihood of the parameters given the observed food web topology. This line of reasoning could provide a means to estimate $\chi$ values based on empirical data, thereby enhancing the model’s applicability to real-world ecosystems. It is crucial to acknowledge, however, that this approach was not within the scope of our present work, and further investigations would be needed to explore the feasibility and validity of such an inference method. As the ecological community advances in data collection and analysis capabilities, efforts to bridge theoretical model parameters with observed food web structures become increasingly pertinent. This potential avenue for extracting $\chi$ from empirical data opens up new opportunities for refining ecological models and aligning them more closely with real-world complexities.

It is important to acknowledge certain limitations in our study. The Extended Niche model we introduced lacks empirical validation, unlike its predecessor, the Niche model by Williams and Martinez (2000), which has been validated using real-world ecological data. Additionally, our model applies the basic ATN approach by Brose et al. (2006) and omits some of the later parameter developments regarding activity and maintenance respiration costs (Boit et al. 2012; Kath et al. 2018) which, however, still lack empirical estimates today. Consequently, our findings are currently confined within a theoretical framework, and future research should prioritize empirical validation to enhance the model’s applicability to real ecosystems. Nonetheless, several of our findings are in line with earlier work (Rall et al. 2008; Rip and McCann 2011), suggesting that the patterns or observations we have identified may have broader applicability and might not be unique to the particular characteristics or assumptions embedded in our model. This careful interpretation implies a level of caution against attributing all observed behaviors solely to the intricacies of our model. Instead, it encourages the recognition that the identified patterns could be indicative of more fundamental and widespread ecological principles. This recognition prompts us to consider the potential relevance of our findings beyond the specific constraints of our model’s assumptions. Nevertheless, to enhance the robustness and real-world applicability of our model, empirical validation remains a crucial step for future research.

In conclusion, our study provides valuable insights into the factors that drive biomass oscillations and stability in complex food webs. Understanding the interplay between consumer interference, metabolic type, food web structure, and diet specialism is crucial for predicting and managing the resilience of ecological communities. These findings contribute to the broader understanding of ecosystem dynamics and can inform conservation and management strategies aimed at preserving biodiversity and ecological stability in natural systems. Future research should explore the mechanisms underlying these observed patterns and their implications for real-world ecosystems.

### Supplementary Information

Below is the link to the electronic supplementary material.

Supplementary file1 (DOCX 2367 KB)

## Data Availability

The data that support the findings of this study are available from the corresponding author upon reasonable request.
