# Supplementary material for: Food-web complexity, consumer behavior, and diet specialism: impacts on ecosystem stability
Source: Theor Ecol. 2024 Apr 25;17(2):131–41. doi: 10.1007/s12080-024-00580-w (PMC11178659; doi:10.1007/s12080-024-00580-w)
Supplement: Supplementary file 1 — Supplementary file1 (DOCX 2367 KB) [file 12080_2024_580_MOESM1_ESM.docx]

**Supporting information for “Food-web complexity, consumer behavior and diet specialism: impacts on ecosystem stability”**

Tommi Perälä^1*^, Mikael Kuisma^2^, Silva ­­Uusi-Heikkilä^1^ and Anna Kuparinen^1^

in ”Theoretical Ecology”

^1^ Department of Biological and Environmental Science, University of Jyväskylä, Finland

^2^ Department of Physics, Technical University of Denmark, Denmark

***Corresponding author**: Tommi Perälä, [tommi.a.perala@jyu.fi](mailto:tommi.a.perala@jyu.fia),

**Additional tables**

**Table S1. Means and medians of the common logarithm of minimum biomass for remaining least abundant species after the removal of extinct species**

|  |  | Ectotherm vertebrates | | | | | Invertebrate predators | | | | |
| --- | --- | --- | --- | --- | --- | --- | --- | --- | --- | --- | --- |
|  | $S$ \ $C$ | 0.10 | 0.15 | 0.20 | 0.25 | 0.30 | 0.10 | 0.15 | 0.20 | 0.25 | 0.30 |
| Mean | 10 | -0.225 | -0.433 | -0.610 | -0.664 | -0.635 | -1.14 | -1.16 | -1.15 | -1.14 | -1.11 |
|  | 20 | -1.22 | -1.48 | -1.46 | -1.29 | -1.11 | -2.00 | -1.93 | -1.78 | -1.63 | -1.49 |
|  | 30 | -2.01 | -2.04 | -1.85 | -1.62 | -1.40 | -2.48 | -2.33 | -2.09 | -1.88 | -1.71 |
| Median | 10 | -3.10 | -3.54 | -3.36 | -3.21 | -2.92 | -3.36 | -3.71 | -3.74 | -3.66 | -3.60 |
|  | 20 | -5.31 | -4.66 | -4.31 | -3.89 | -3.28 | -6.21 | -5.52 | -5.07 | -4.66 | -4.12 |
|  | 30 | -5.93 | -5.50 | -5.07 | -4.56 | -3.81 | -6.84 | -6.16 | -5.76 | -5.21 | -4.54 |

**Additional Figures**


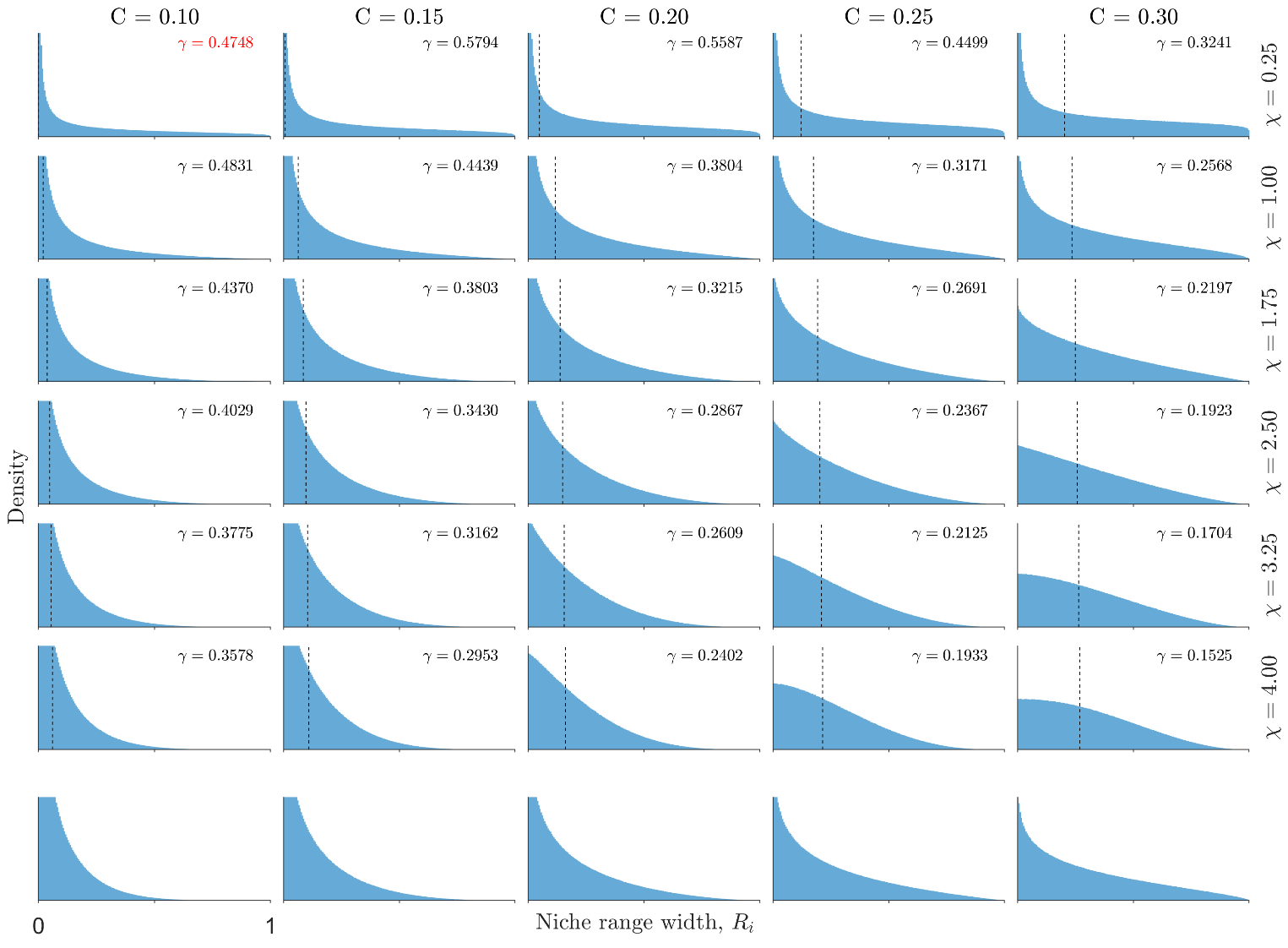


**Fig S1** Distribution of niche range widths $(R_{i})$ in the Extended Niche model for various $(C,\chi)$ pairs. The blue histograms depict the distribution of niche range widths, generated from 10 million random draws. Each column corresponds to different values of network connectance $C\in\{0.10, 0.15, 0.20, 0.25, 0.30\}$, and the top six rows illustrate the impact of the new parameter $\chi\in\{0.25, 1.00, 1.75, 2.50, 3.25, 4.00\}$. Dashed black lines represent the median of the distributions, consistently shifting to the right with increasing $\chi$ values. Nonparametric skewness $\gamma$, calculated as the difference between the mean and the median divided by the standard deviation, also consistently decreases with $\chi$ (except for one outlier, highlighted in red font). The bottom row displays the distribution of $R_{i}$ for the original Niche model, providing a basis for comparison.


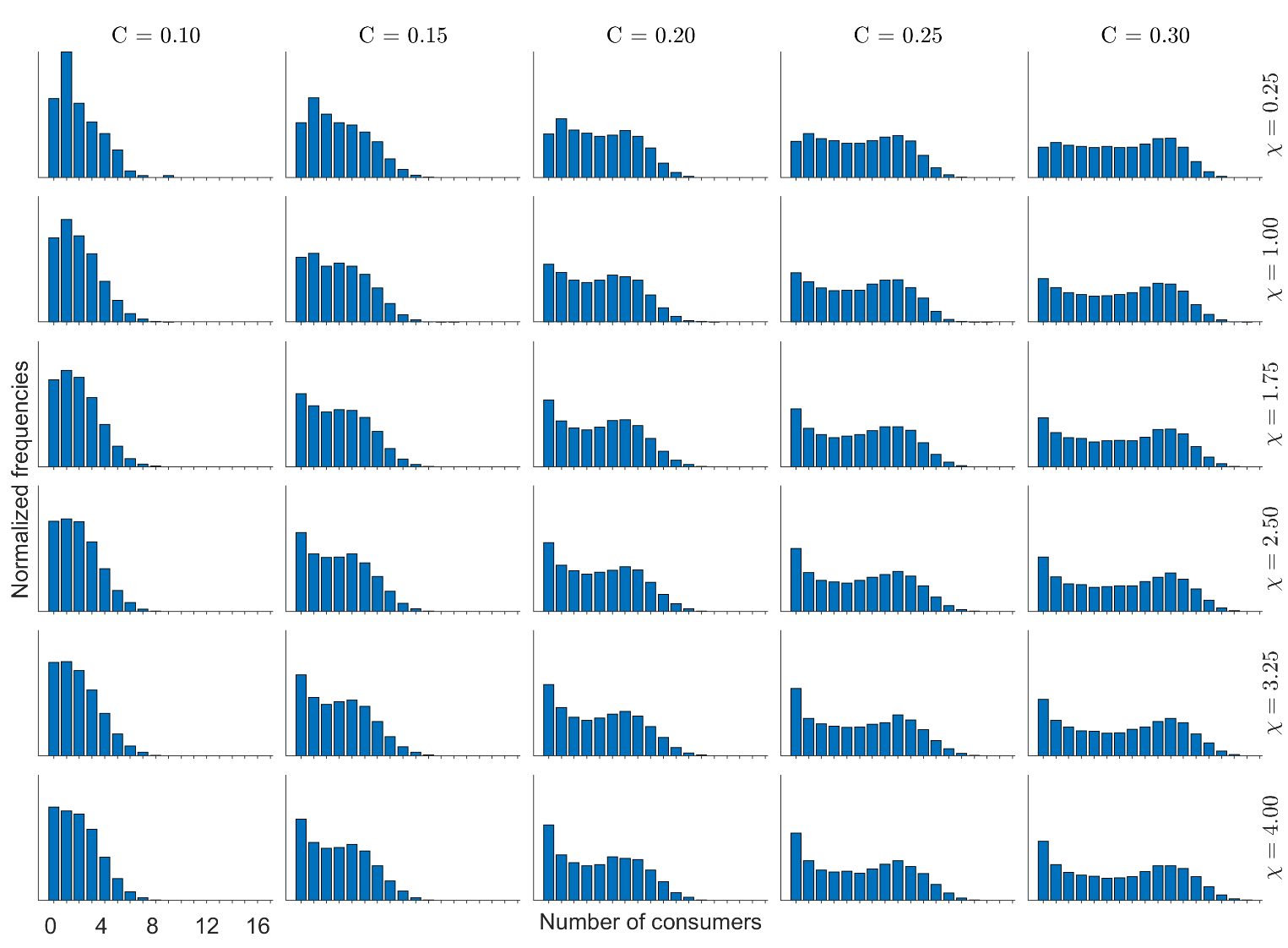


**Fig S2** Impact of $\chi$ on the in-degree distribution (vulnerability) in the Extended Niche model. Each column represents a distinct value of network connectance $(C)$, and rows depict the effects of the new parameter $\chi$ on vulnerability. Blue histograms show the distribution of in-degree values. The effect of $\chi$ on the in-degree distribution is minor and is almost completely masked by the effect of $C$. The data is presented for food webs of size $S=20$. Food webs with $S=10$ and $S=30$ exhibit almost identical shaped distributions and are therefore not shown.


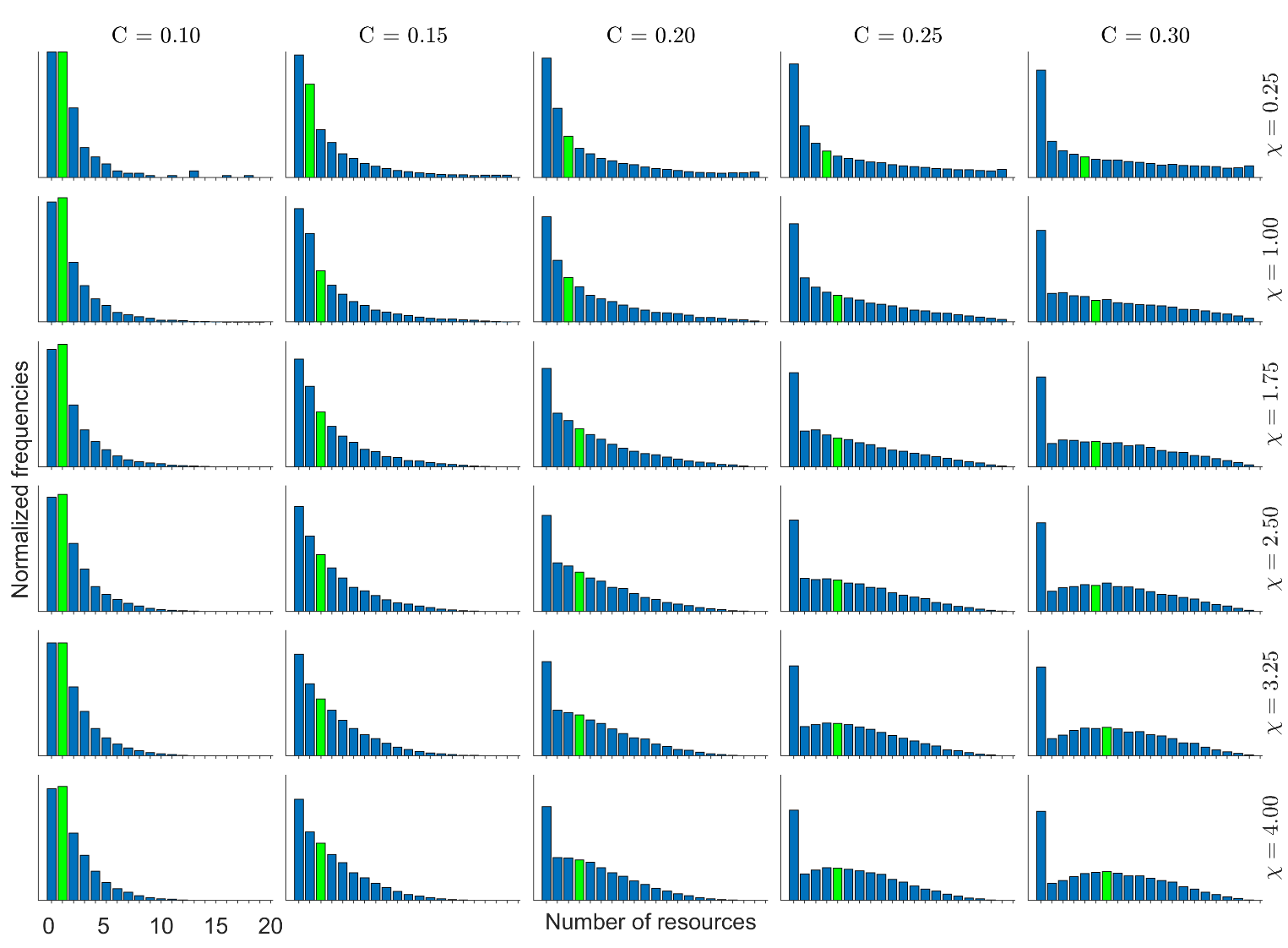


**Fig S3** Influence of $\chi$ on the out-degree distribution (generality) of the generated food webs in the Extended Niche model. Columns represent different values of network connectance $(C)$, while rows showcase the impact of the parameter $\chi$ on the out-degree. Blue histograms depict the distribution of out-degree values, and the green bar represents the median of the distribution. An increase in $\chi$ leads to a slight increase in the median, with a more pronounced effect observed for food webs with higher connectance. The data is presented for food webs of size $S=20$. Food webs with $S=10$ and $S=30$ exhibit almost identical shaped distributions and are therefore not shown.


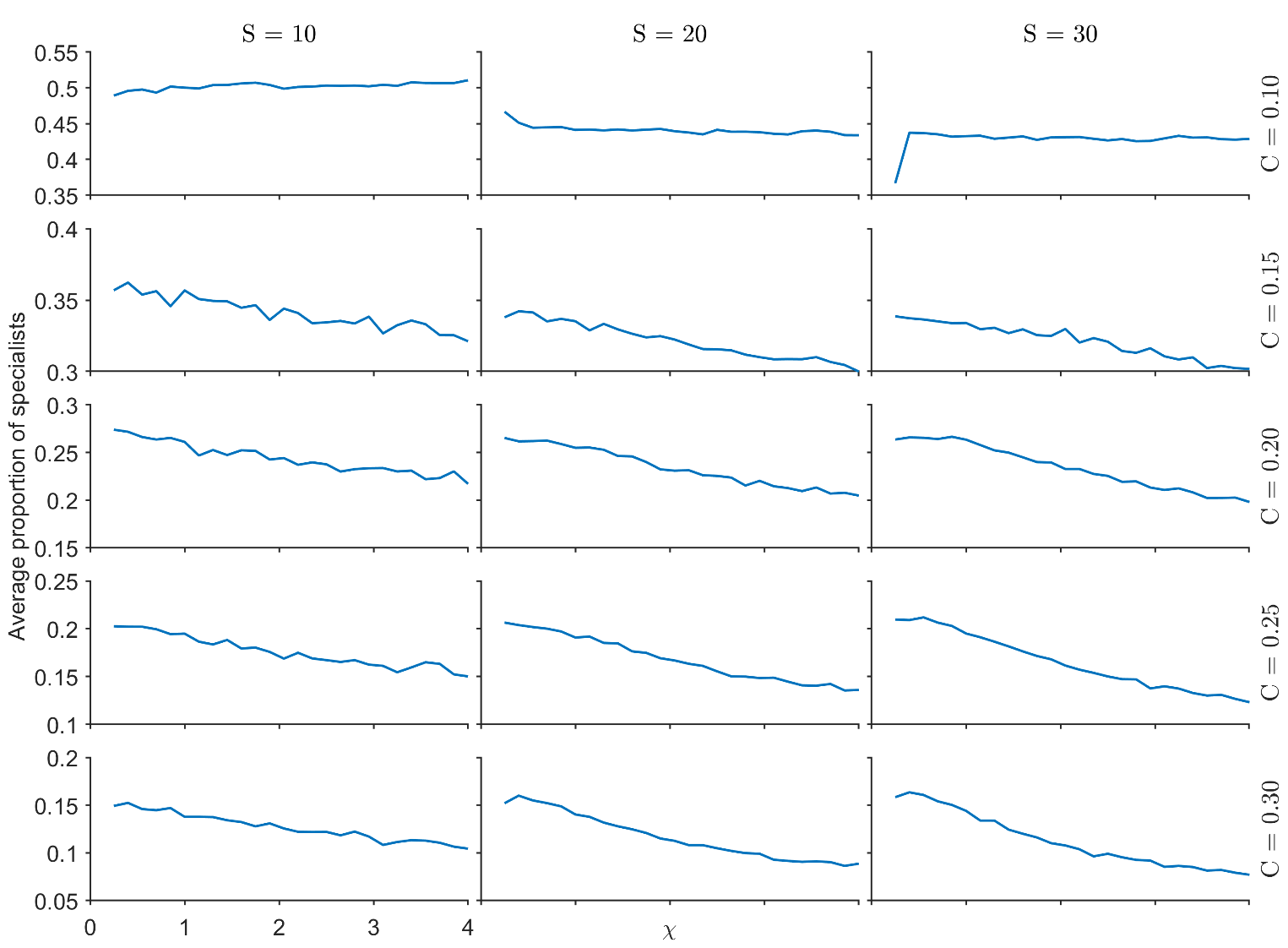


**Fig S4** Exploring the relationship between the average prevalence of specialists, defined in our work as having at least one but most $0.1S$ resource species, and the parameter $\chi$ in food webs generated by the Extended Niche model. Columns represent different numbers of species $(S)$ and rows showcase various levels of network connectance $(C)$. Each panel illustrates how the average proportion of specialists varies with the parameter $\chi$. In all but the lowest connectance category, increasing $\chi$ decreases the number of specialists. Note the outliers in the top row, attributed to the problematic corner of the parameter space (Table 1).


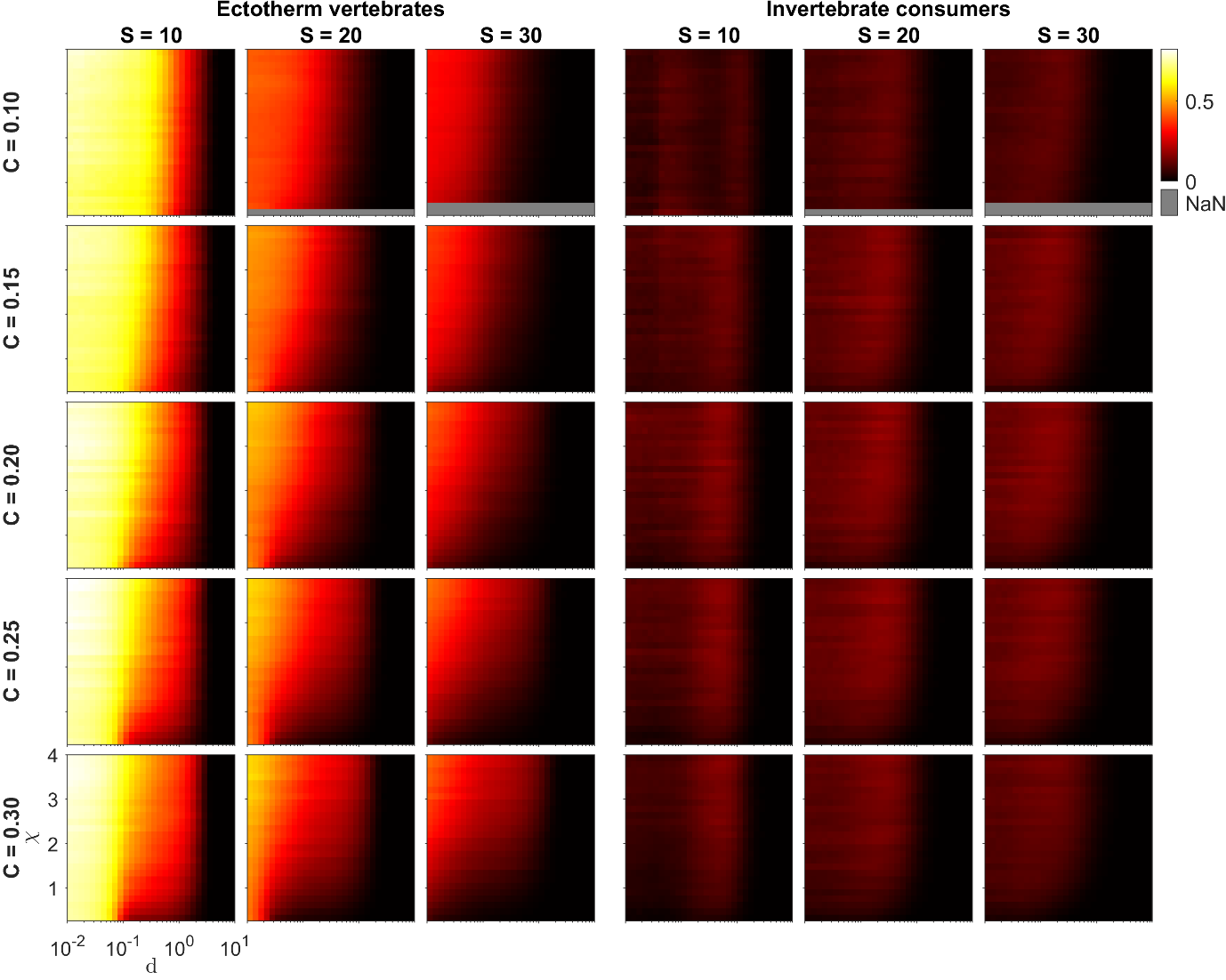


**Fig S5** Food-web oscillation measure $O_{\mathrm{tot}}$ as a function of the second Extended Niche model parameter $\chi$ and intraspecific consumer interference parameter $d$ for small, intermediate and large network sizes $S$, five values of network connectance $C\in\{0.10, 0.15,0.20,0.25,0.30\}$, and two metabolic types of consumers (ectotherm vertebrates, invertebrate consumers). The lighter the color the higher the average $O_{\mathrm{tot}}$ for the food webs generated with the corresponding parameter value 5-tuple $(\mathrm{MType}, S,C,\chi, d)$. For the figure, we filtered out those parameter combinations that had less than 300 replicate food webs

**
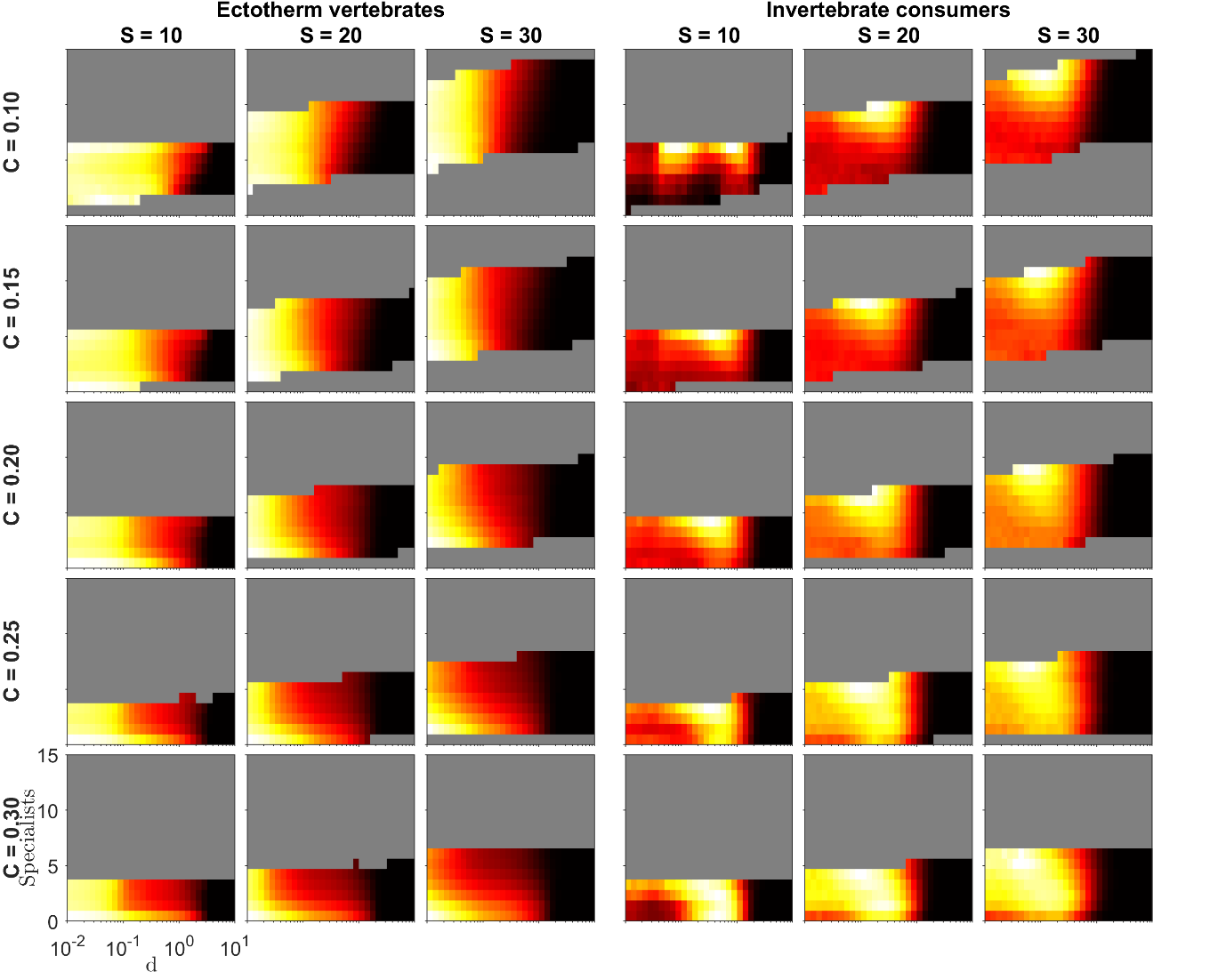
**

**Fig S6** Food-web oscillation measure $O_{\mathrm{tot}}$ as a function of the number of specialists and intraspecific consumer interference parameter $d$ for small, intermediate and large network sizes, five values of network connectance $C\in\{0.10, 0.15,0.20,0.25,0.30\}$, and two types of consumers (ectotherm vertebrates, invertebrate consumers). The lighter the color the higher average $O_{\mathrm{tot}}$ for the corresponding food-web property – food-web dynamics parameter value pair $(\mathrm{Specialists}, d)$. A species was considered a specialist when it had at most 10% of the species in the original food web on its diet. Each panel has its own scale, and as the figure attempts to highlight the relative effect of specialism within each panel, the color bars are not shown. Moreover, only those $(Specialists, d)$ pairs are shown that had data from at least 700 simulations. Furthermore, for aesthetic reasons, a robust mean estimator was used, which ignored outliers whose distance to the mean was more than 4 standard deviations

**
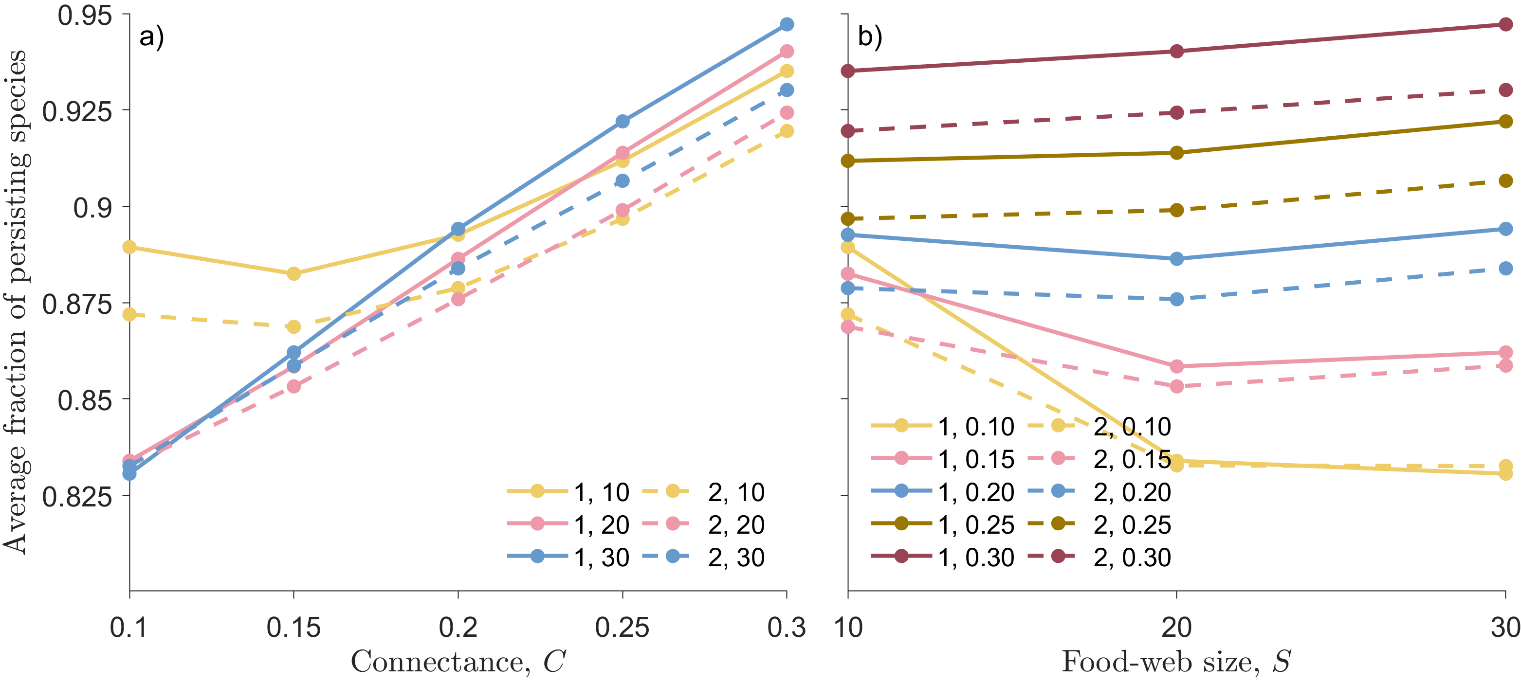
Fig S7** Average fraction of persisting species in the food webs generated randomly using the Extended Niche model, $\mathrm{NICHE}_{3}(S,C,\chi)$ plotted against connectance (panel a) and food-web size (panel b). The line style and color denote different pairs of metabolic type (1 = ectotherm vertebrates, 2 = invertebrate consumers) and $S$ (panel a), and metabolic type and $C$ (panel b)
